# Cardiogenic Shock Due to Serotonin Syndrome Induced Takotsubo Cardiomyopathy

**DOI:** 10.7759/cureus.38595

**Published:** 2023-05-05

**Authors:** Vahid Yazdi, Zachary Cox, Mitra Patel, Bayan Yazdi, Paul Chacko

**Affiliations:** 1 Department of Cardiovascular Medicine, The University of Toledo College of Medicine and Life Sciences, Toledo, USA; 2 Department of Cardiology, Loyola University Chicago Stritch School of Medicine, Maywood, USA; 3 Department of Cardiovascular Medicine, The University of Toledo Medical Center, Toledo, USA

**Keywords:** critical care, cardiology, serotonin syndrome, takotsubo cardiomyopathy, cardiogenic shock

## Abstract

Takotsubo cardiomyopathy causes transient left ventricular dysfunction. It typically has a favorable prognosis but rarely leads to complications such as cardiogenic shock. Also known as stress-induced cardiomyopathy, it is precipitated by emotional or physical stress. Serotonin syndrome can cause severe stress due to excessive serotonergic activity in the central nervous system. We report a case of cardiogenic shock precipitated by serotonin syndrome-induced takotsubo cardiomyopathy. Only one other documented case has exhibited cardiogenic shock in this setting.

## Introduction

Takotsubo cardiomyopathy (TCM), or stress-induced cardiomyopathy, is characterized by transient, reversible left ventricular (LV) dysfunction due to apical ballooning without coronary artery obstruction. It is commonly seen in post-menopausal females following significant emotional or physical stress [1]. Its pathophysiology remains unclear, but the two most supported current suggestions are due to microvascular dysfunction and elevated catecholamine-induced myocardial toxicity [2,3]. Although the prognosis is typically favorable and the condition is reversible, many complications such as cardiogenic shock, arrhythmias, pulmonary edema, and LV thrombus are associated with TCM [4]. In the clinical presentation, electrocardiographic (ECG) findings, and biomarker profiles, TCM presents similarly to acute coronary syndrome (ACS). However, it is distinguishable by regional wall abnormalities extending beyond a coronary vascular bed, the absence of coronary artery occlusion, and is preceded by a precipitating factor [5].

Serotonin syndrome is a life-threatening condition caused by excess serotonergic activity in both the peripheral and central nervous system [6]. It is typically precipitated by medication combinations that increase serotonin levels. Classically, it presents with a triad of altered mental status, autonomic nervous system overactivity, and neuromuscular hyperactivity [6]. Serotonin syndrome has been associated with takotsubo cardiomyopathy, but only one other reported case has presented with cardiogenic shock [7]. Here, we present a 41-year-old female who developed cardiogenic shock following TCM caused by serotonin syndrome.

## Case presentation

A 41-year-old female with a past medical history significant for myocardial infarction, type 2 diabetes, paraplegia of the lower extremities secondary to meningitis, bipolar disorder, and chronic obstructive pulmonary disease presented to the emergency department (ED) with intermittent sharp chest pain and mild disorientation with chills and a headache.

**Figure 1:**
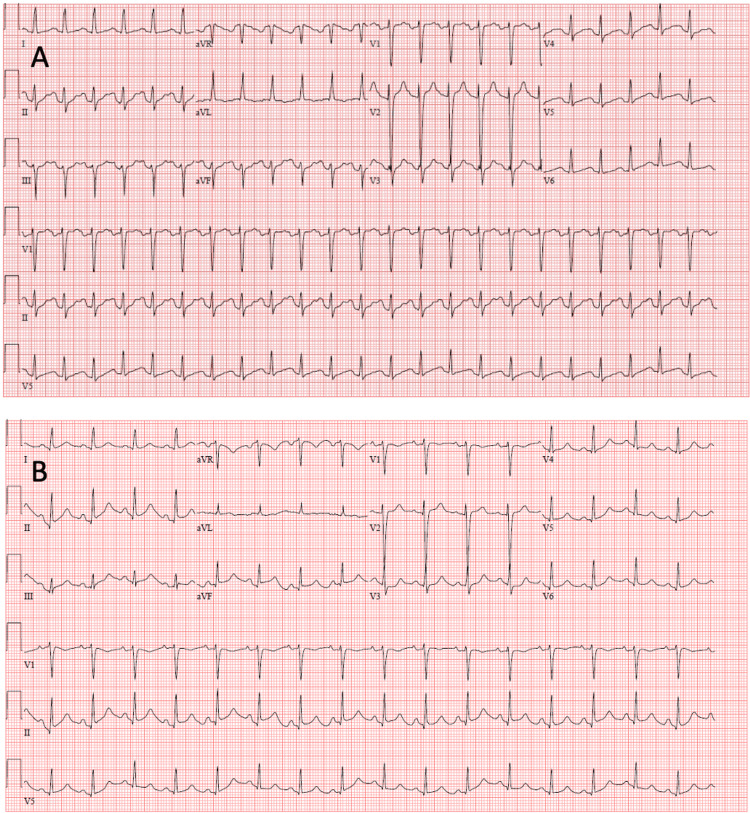
(A) Initial electrocardiogram shows nonspecific T wave abnormalities in the inferolateral distribution. (B) Repeat electrocardiogram shows resolution of these abnormalities.

In the ED, the patient was afebrile with a blood pressure of 91/45 mm Hg, a heart rate of 147 beats per minute (BPM), and an O2 saturation of 98% on 3 L of O2 via nasal cannula. After the morphine infusion, her heart rate improved to 94 bpm and her blood pressure to 159/118 mm Hg. Initial ECG revealed nonspecific T wave abnormalities in the inferolateral distribution (Figure 1A). Troponin was elevated at 0.44 ng/mL. A chest X-ray showed a small right pleural effusion. CT angiography of the chest showed small bilateral effusions with mixed interstitial and airspace pulmonary edema without evidence of pulmonary embolism. Early the next morning, the patient developed a temperature of 103.2°F and then became suddenly hypotensive with a systolic blood pressure of 60 mmHg. Lactate was elevated at 3.5 mmol/L, and troponin was elevated to 2.71 ng/mL and trended to 4.87 ng/mL. A repeat ECG showed normal sinus rhythm with no abnormalities (Figure 1B).

**Figure 2:**
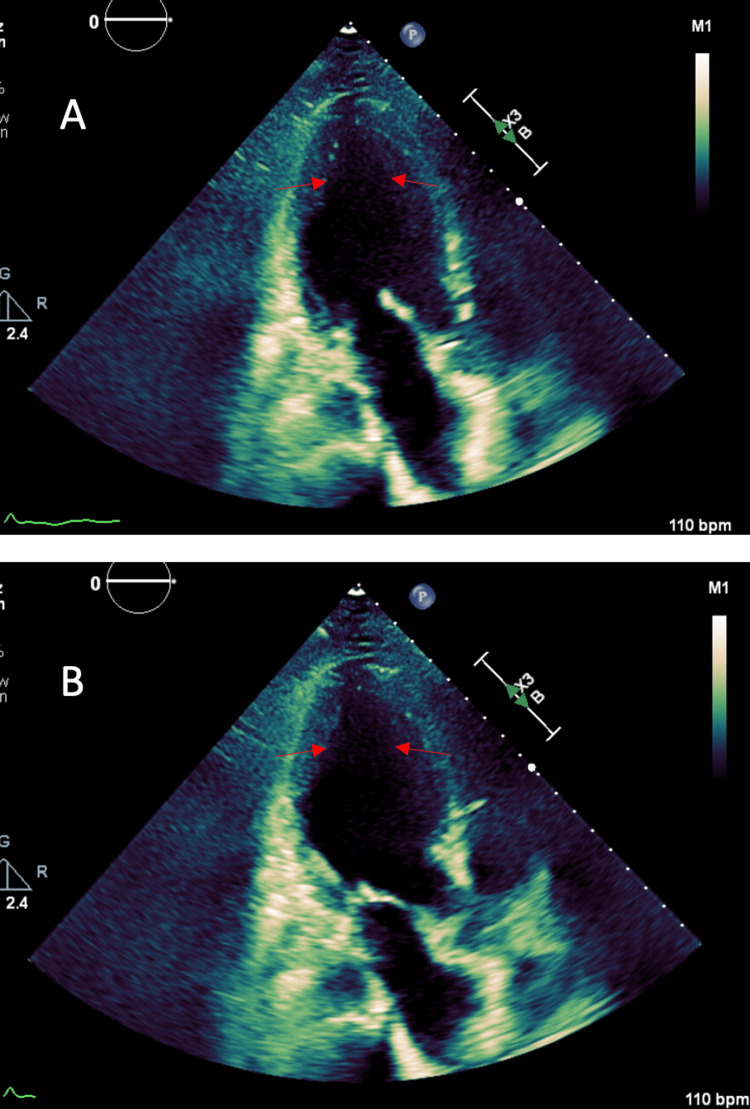
Echocardiography in (A) end-diastolic phase and (B) end-systolic phase on admission. Arrows point to ventricular wall abnormalities.

The patient was transferred to the intensive care unit (ICU) and placed on mechanical ventilation and norepinephrine drip for acute respiratory failure and lactic acidosis. Linezolid and meropenem were started due to the patient’s multidrug-resistant organism urinary tract infection history and vancomycin allergy. Central venous pressure was elevated at 22-24 mm Hg and mixed venous O2 was low at 49%. Bedside transthoracic echocardiography (TTE) showed a left ventricular ejection fraction (LVEF) of 5-10% with global hypokinetic and akinetic ventricular wall segments (Figure 2A, 2B). The temperature spiked to 106.9°F. The patient’s white blood cell count, CT of the chest, abdomen, and pelvis, and urinalysis were unremarkable for infection. Right and left heart catheterizations were normal.

The patient's medication list included mirtazapine, escitalopram, sumatriptan, and aripiprazole. Following the withdrawal of serotonergic medications, her condition began to improve, antibiotics were stopped, and she was extubated. The patient later reported having a migraine prior to the presentation and being given “new migraine medication” at her skilled nursing facility, but it is unknown what she was given.

Escitalopram, sumatriptan, and aripiprazole were restarted two weeks later with no adverse effects. Mirtazapine was discontinued.

**Figure 3:**
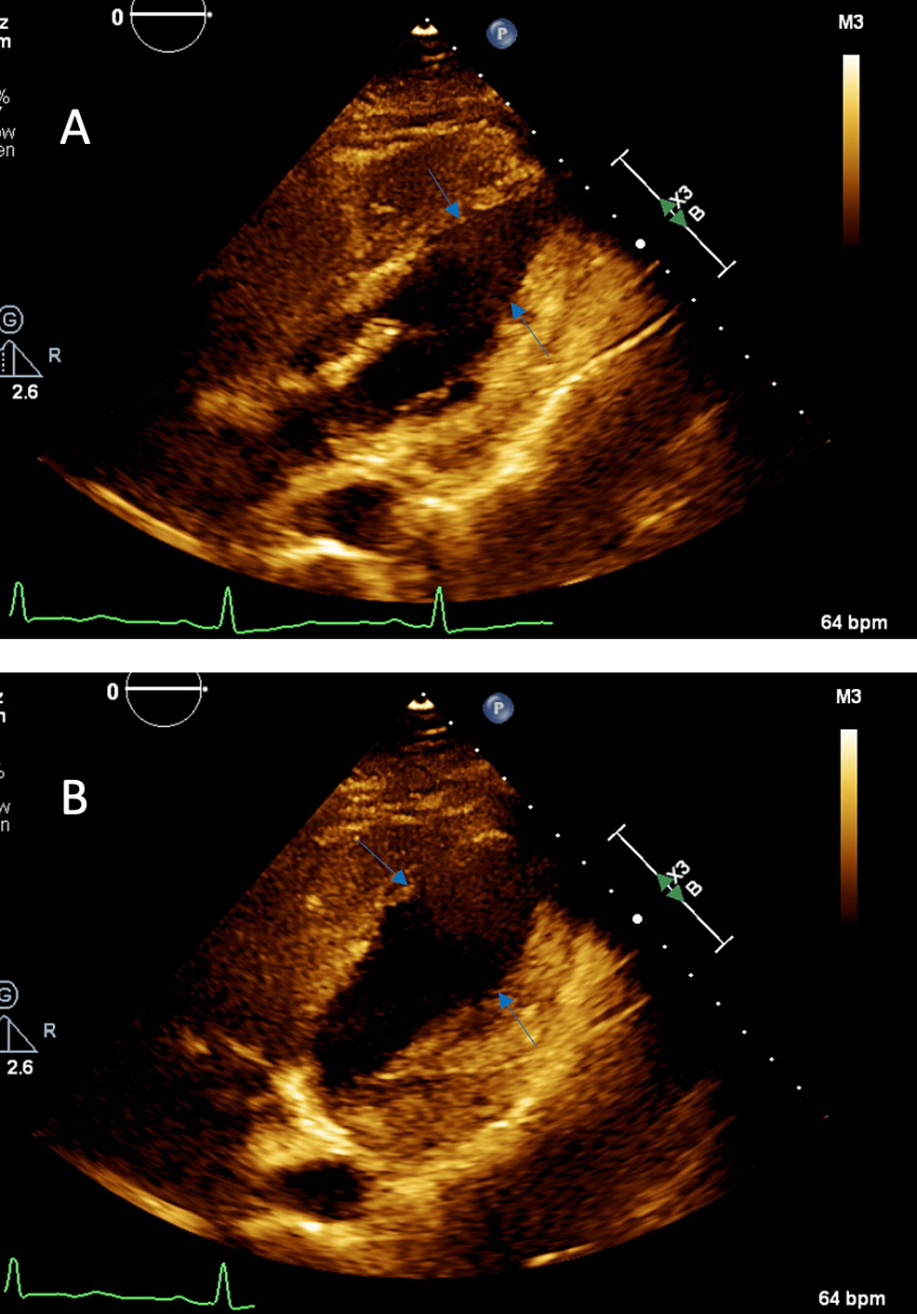
Two-month echocardiography follow-up in (A) end-diastolic phase and (B) end-systolic phase. Arrows point to resolution of ventricular wall abnormalities.

A TTE performed two months after discharge found no regional wall motion abnormalities and an LVEF of 55% (Figure 3A, 3B).

## Discussion

We detail a patient with TCM causing cardiogenic shock likely following ingestion of multiple serotonergic-acting medications. The underlying etiology of TCM includes excessive catecholamine-induced myocardial toxicity. Though it is reversible and has a generally favorable prognosis, it may lead to serious complications such as cardiogenic shock, which has been found in 4.2% of cases [7].

Although there are no direct laboratory tests to definitively confirm serotonin syndrome, many criteria have been proposed for diagnosis. Among these is the Radomski criteria, which require at least four major symptoms or three major symptoms with two minor symptoms (major symptoms include confusion, elevated mood, coma or semi-coma, fever, hyperhidrosis, myoclonus, tremors, chills, rigidity, and hyper-reflexia; minor symptoms include agitation, insomnia, tachycardia, tachypnea, diarrhea, low or high blood pressure, impaired coordination, mydriasis, and akathisia) [8]. Our patient fulfilled three major and two minor symptoms, confirming the diagnosis of serotonin syndrome. However, the specific contributing medication could not be accurately identified as she was on multiple serotonergic and dopaminergic-acting agents (mirtazapine, escitalopram, aripiprazole, sumatriptan, and linezolid). All serotonergic medications were discontinued following stabilization, and her condition gradually improved.

The hyperadrenergic state caused by serotonin syndrome is consistent with the preceding emotional or physiologic trigger seen in the majority of TCM occurrences. The mechanism by which a hyperadrenergic state could lead to TCM is described as “stimulus trafficking.” [9] In this process, excess levels of catecholamine induce ß-2 coupling from Gs to Gi to protect myocytes from strong Gs overstimulation. This results in hypokinesia with the greatest involvement at the apex due to higher adrenoceptor density at the apex than the base, along with slow increases in serum troponin levels reflecting early minimal necrosis. These findings were observed in our patient, who had akinetic and hypokinetic walls on TTE along with a markedly elevated troponin and the absence of coronary artery occlusion on catheterization. Additionally, the resolution of our patient’s wall motion abnormalities with normalization of her LVEF two months after this episode further suggests TCM, as it is transient and reversible. In the setting of her hemodynamic instability, elevated central venous pressure (CVP), and low mixed venous O2 with these echocardiographic findings, cardiogenic shock was diagnosed.

## Conclusions

Though the association between TCM and serotonin syndrome has been reported multiple times, it has rarely been seen to lead to cardiogenic shock, as in our patient. It is important to be cognizant of serotonin syndrome-induced TCM and the ensuing cardiogenic shock when managing patients on serotonergic and dopaminergic agents.

## References

[1] Templin C, Ghadri JR, Diekmann J (2015). Clinical features and outcomes of takotsubo (stress) cardiomyopathy. N Engl J Med.

[2] Sato H, Tateishi H, Uchida T (1990). Takotsubo-type cardiomyopathy due to multivessel spasm. Clinical Aspect of Myocardial Injury: From Ischemia to Heart Failure.

[3] Schneider B, Athanasiadis A, Schwab J (2014). Complications in the clinical course of tako-tsubo cardiomyopathy. Int J Cardiol.

[4] Hurst RT, Prasad A, Askew JW 3rd, Sengupta PP, Tajik AJ (2010). Takotsubo cardiomyopathy: a unique cardiomyopathy with variable ventricular morphology. JACC Cardiovasc Imaging.

[5] Scotton WJ, Hill LJ, Williams AC, Barnes NM (2019). Serotonin syndrome: pathophysiology, clinical features, management, and potential future directions. Int J Tryptophan Res.

[6] Sasaki H, Yumoto K, Nanao T, Nishizawa H, Funada S, Aoki H, Kato K (2013). Cardiogenic shock due to takotsubo cardiomyopathy associated with serotonin syndrome. J Cardiol Cases.

[7] Gianni M, Dentali F, Grandi AM, Sumner G, Hiralal R, Lonn E (2006). Apical ballooning syndrome or takotsubo cardiomyopathy: a systematic review. Eur Heart J.

[8] Radomski JW, Dursun SM, Reveley MA, Kutcher SP (2000). An exploratory approach to the serotonin syndrome: an update of clinical phenomenology and revised diagnostic criteria. Med Hypotheses.

[9] Lyon AR, Rees PS, Prasad S, Poole-Wilson PA, Harding SE (2008). Stress (takotsubo) cardiomyopathy--a novel pathophysiological hypothesis to explain catecholamine-induced acute myocardial stunning. Nat Clin Pract Cardiovasc Med.

